# Alkali Activation of Copper and Nickel Slag Composite Cementitious Materials

**DOI:** 10.3390/ma13051155

**Published:** 2020-03-05

**Authors:** Tingting Zhang, Shiwei Zhi, Tong Li, Ziyu Zhou, Min Li, Junnan Han, Wenchen Li, Dan Zhang, Lijie Guo, Zhenlin Wu

**Affiliations:** 1 Faculty of Infrastructure Engineering, Dalian University of Technology, Dalian 116023, China; tingtingzhang@dlut.edu.cn (T.Z.); dlutzsw@mail.dlut.edu.cn (S.Z.); tongtongli@mail.dlut.edu.cn (T.L.); zou_jing8@mail.dlut.edu.cn (Z.Z.); wangzzz777@mail.dlut.edu.cn (M.L.); junnanhan@dlut.edu.cn (J.H.); 2 National Centre for International Research on Green Metal Mining, BGRIMM Technology Group, Beijing 102628, China; liwenchen@bgrimm.com (W.L.); zhangdan@bgrimm.com (D.Z.); guolijie@bgrimm.com (L.G.); 3 School of Optoelectronic Engineering and Instrumentation Science, Dalian University of Technology, Dalian 116023, China

**Keywords:** copper and nickel slag, alkali-activated, cementing material, geopolymer

## Abstract

Alkali-activated copper and nickel slag cementitious materials (ACNCMs) are composite cementitious materials with CNS (copper and nickel slag) as the main materials and GGBFS (ground-granulated blast-furnace slag) as a mineral admixture. In this paper, the activity indexes of CNS with different grinding times were studied using CNS to replace a portion of cement. NaOH, Na_2_SO_4_, and Na_2_SiO_3_ activators were used to study the alkaline solution of the CNS glass phase. The effects of the fineness of CNS and the type of activator on the hydration of ACNCMs were investigated via physical/mechanical grinding and chemical activation. The hydration products of ACNCMs were analyzed via XRD, SEM, FT-IR, TG, and MIP. The results of the study revealed that the activity indexes of CNS ground with different grinding times (10, 30 and 50 min) were 0.662, 0.689, and 0.703, respectively. When Na_2_SiO_3_ was used as the activator, the glass phase dissolved the most Si^4+^, Al^3+^, and Ca^2+^, and the respective concentrations in the solution were found to be 2419, 39.55, and 3.38 mg/L. Additionally, the hydration products of ACNCMs were found to have a 28-day compressive strength of up to 84 MPa.

## 1. Introduction

Nonferrous slags are an industrial by-product, most of which are treated by stacking or landfill. Since these smelting slags contain many heavy metals, such as copper, nickel, cobalt, and zinc, a large amount of industrial smelting slag accumulation or landfill not only occupies farmland and other lands, but it also seriously endangers the environment [1,2].

Nickel slag is the waste residue discharged during the smelting of nonferrous nickel metals [3]. For every ton of nickel produced, 6–16 tons of nickel slag are produced [4,5]. According to statistics, the annual discharge of nickel slag in China is 81.05 million tons. Nickel slag is mainly used in the recovery of valuable metal, glass manufacturing, and ceramic production, among other applications [6,7,8]. In recent years, research has been conducted on the use of nickel slag to prepare cementitious materials or as a substitution for fine aggregate sand in concrete [9,10]. To reduce the cost of nickel production, molten tailings are poured directly in the factory, and the tailings are slowly cooled by air. Therefore, the content of the glass phase in nickel slag is low, and its activity is poor, which leads to the low utilization rate (only 8%) of nickel slag [11,12].

Geopolymers are a cementitious material produced by the reaction of solid aluminum silicate materials with high concentrations of an activator solution. These activators usually include alkali metal hydroxides and silicates [13], and aluminum silicate materials are mainly fly ash, metakaolin, slag, steel slag, and copper slag [14,15,16,17,18]. The use of activators is an effective way to prepare geopolymers from smelting slag for engineering filling while improving the utilization rate, reducing the production cost, and reducing the greenhouse gas emission [19,20,21,22].

Yang et al. [20] prepared cementing materials for mine filling with nickel slag as the main material, and sodium sulfate, desulfurization gypsum, calcium carbide slag, and cement clinker as the activators. A study by Yang [23] revealed that geopolymers prepared by high magnesia-nickel slag instead of 20% fly ash have a denser structure, higher compressive strength, and lower shrinkage. By optimizing the content of a sodium silicate activator, the compressive strength of a high-magnesium nickel slag and fly ash polymer can reach up to 60 MPa [1]. Lan [24] prepared a new type of low-strength filling material; where lime, sodium hydroxide, triethanolamine, and finely-ground copper slag were mixed fully into a slurry with a 70% solid mass fraction according to the ratio of 18:2:0.1:100. The 28-d compressive strength of the new filling material was found to reach up to 1.0 MPa, and, therefore, can be used for underground filling. Singh [25] prepared alkali-activated cementitious material; where copper slag, sodium silicate solution (Ms = 3.3, SiO_2_ = 26.5%, Na_2_O = 8%, H_2_O = 65.5%), sodium hydroxide, water, and sand were fully mixed according to the ratio of 1000: 330: 56: 135: 3000. The 28-d compressive strength of the cementitious material was found to reach up to 34.12 MPa.

The contents of silicon, aluminum, and calcium in the smelting slags studied by these scholars were found to be relatively high with more active components. However, the preparation of geopolymers from CNS (copper and nickel slag) with fewer active components has been rarely reported. Large quantities of CNS have been piled up in Xinjiang, China, causing serious environmental pollution. The most effective method of absorbing solid waste and making full use of the waste residue is to prepare CNS into a geopolymer and fill it into mines. In this study, geopolymer ACNCMs (alkali-activated copper and nickel slag cementitious materials), which have a high compressive strength, were prepared with CNS as the main material and GGBFS (ground-granulated blast-furnace slag) as the mineral admixture using two methods of mechanical activation and chemical excitation, wherein the utilization rate of waste slag was found to be high. This outcome provides a basis for the filling of mines in Xinjiang.

## 2. Materials and Methods

### 2.1. Materials

The main material of the ACNCMs used in this study was CNS from the Karatongke mine in Xinjiang. Table 1 lists the chemical compositions of CNS and GGBFS. CNS mainly contains iron and silicon (${\mathrm{SiO}}_{2}$ and ${\mathrm{Fe}}_{2}O_{3}$ account for 86%). However, the calcium oxide content and aluminum oxide content are very small, only 1.66% and 1.18%, respectively. The mineral admixture is composed of S95 slag powder. GGFBS mainly contains alumina, silica, and calcium oxide, of which calcium oxide is the most abundant, accounting for nearly 50%.

**Figure 1:**
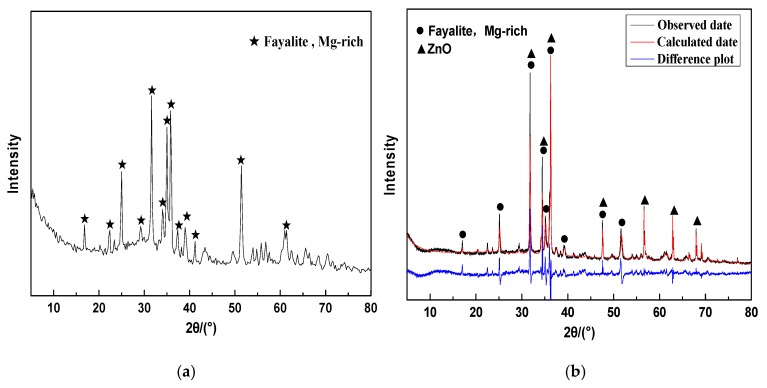
(**a**) XRD spectrum and (**b**) Rietveld full-spectrum fitting results of CNS.

Figure 1a presents the XRD (X-ray diffraction) pattern of CNS, which is mainly composed of a crystalline phase and an amorphous glass phase. The main crystalline material is fayalite containing magnesium (Fe,Mg)_2_SiO_4_. Figure 1b presents the Rietveld full-spectrum fitting results of CNS. Table 2 exhibits the results of the quantitative analysis of CNS, from which it is evident that CNS is composed of a 72.42% fayalite crystalline phase and 27.58% glass phase.

The activators were sodium hydroxide (NaOH), sodium sulfate (${\mathrm{Na}}_{2}{\mathrm{SO}}_{4}$), and anhydrous sodium silicate (${\mathrm{Na}}_{2}{\mathrm{SiO}}_{3}$). These chemicals were analytically pure.

### 2.2. Methods

#### 2.2.1. Activity Index of CNS

The activity index of CNS was calculated according to the national industry standard JG/T 315–2011, the “Natural Volcanic Matter Materials for Cement Mortar and Concrete.” The specimens used in the experiment were divided into a control group and an experimental group. The control group was 100% cement with a 0.5 water/binder ratio and a 1:3 cement/sand ratio, and the experimental group was 70% cement and 30% CNS with a 0.5 water/binder ratio and a 1:3 cement/sand ratio. The cement used in the experiment was CEM I 52.5 R. The prepared specimens were put into water for curing to various ages, and the activity of CNS was evaluated using the ratio of the compressive strengths of the two groups of specimens.

#### 2.2.2. Crystalline Phase and Glass Phase Spatial Structures of CNS

Nuclear magnetic resonance analysis and Raman analysis are common methods for the analysis of the spatial structure of matter. Given the high content of ${\mathrm{Fe}}_{2}O_{3}$ in CNS, it has a certain magnetism; thus, the applicability of nuclear magnetic analysis is limited. Therefore, the spatial structures of the crystalline and glass phases in CNS were analyzed using Raman analysis.

#### 2.2.3. Alkali Dissolution in Glass Phase

A study on the alkaline dissolution characteristics of the CNS glass phase was conducted by Li [26]. First, 1 g of CNS was weighed and respectively soaked in 100 mL of 1 mol/L NaOH, ${\mathrm{Na}}_{2}{\mathrm{SO}}_{4}$, and ${\mathrm{Na}}_{2}{\mathrm{SiO}}_{3}$ solutions. The temperature was controlled within 20 ± 1 °C. After 3 days, the solution was centrifuged for 20 min at a frequency of 3000 r/min. Then, 5 mL of supernatant was taken for analysis, and the concentrations of Si, Al, and Ca plasma were measured by an inductively coupled plasma (ICP) spectrometer.

#### 2.2.4. Preparation of Samples

To improve the activity of CNS and promote its hydration reaction, we used a planetary ball mill to mechanically grind CNS for 10, 30, and 50 min, respectively. Next, 70% of ground CNS was mixed with 30% mineral powder, and the chemical activators NaOH, ${\mathrm{Na}}_{2}{\mathrm{SO}}_{4}$, and ${\mathrm{Na}}_{2}{\mathrm{SiO}}_{3}$ were then respectively weighed. The weights were 7% of the mass of the cementitious material (70% CNS and 30% GGFBS). Water was weighed according to the water-gel ratio of 0.23, and the weighted chemical activators were dissolved respectively in the water. The cementitious material and water were then poured into the mixer for mixing. Finally, the stirred pastes were poured into 20×20×20 mm molds. After vibration compaction (120 s), the pastes were placed into a curing box with a temperature of 20 ± 1 °C and a humidity of more than 90% for 1 d, and they remained in the curing box after mold removal until the corresponding ages were reached.

#### 2.2.5. Test

A Shanghai Hualong WHY-300/10 microcomputer experimental press was used to test the compressive strengths of the test samples. The reference specification was GB/T 17671-1999, the “Test Method for Strength of Cement Glue Sand.” Qualitative analysis of the original sample and hydration products of the CNS was conducted using an X-ray diffractometer (XRD; D8 Advance AXS, Brooklyn, Germany) with a Cu target, a working voltage of 40 kV, a working current of 40 mA, a scanning range of 5–80° (2θ), a scanning step size of 0.02°, and a step time of 0.5 s. For quantitative analysis, the step time was adjusted to 1 s. The instrument used for the analysis of chemical bonds and functional groups was a Bruker EQUINOX55 Fourier-transform infrared spectrometer from Germany, which had a spectral range of 400–4000 cm^−1^. A NOVA Nano SEM 450 from the FEI Company in the United States was used for the analysis of the micro-morphologies of the samples. The X-ray fluorescence spectrometer was an XRF-1840 model from Japan. The instrument used for the alkaline dissolution of the glass phase of CNS was a plasma emission spectrometer (Optima2000DV, Waltham, MA, USA).

## 3. Results and Discussion

### 3.1. Activity Index of CNS

**Figure 2:**
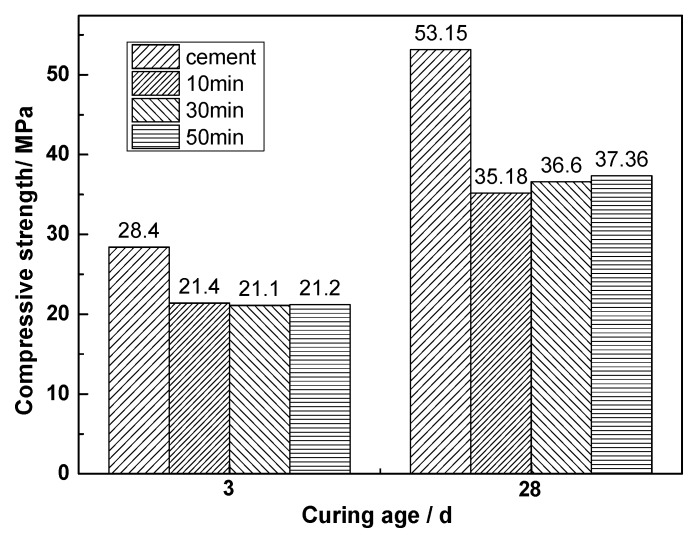
Strength histogram of the 30% replacement of cement with CNS.

Figure 2 is the histogram of the compressive strengths of the hardened bodies after the replacement of CEM I 52.5 R cement with 30% CNS of different grinding times. The addition of CNS was found to reduce the compressive strength of cement sand. In the early stage of cement hydration, the grinding time of CNS was found to have little effect on the cement mortar, and the strength of the cement mortar gradually increased with the increase of grinding time at 28 days of hydration. The 28-day activity index of CNS was calculated according to Equation (1). The activity indexes of CNS ground for 10, 30, and 50 min were 0.662, 0.689, and 0.703, respectively. It is evident that CNS with a high iron content has certain potential activity, and that mechanical grinding can improve the activity of CNS.

(1) $$A_{28}=\frac{R_{28}}{R_{028}},$$

where *R*_28_ is the 28-day compressive strength of the experimental group, and *R*_028_ is the 28-day compressive strength of the control group.

$$A_{28}10\min=\frac{35.18}{53.15}=0.662;\;A_{28}30\min=\frac{36.6}{53.15}=0.689;\;A_{28}50\min=\frac{37.36}{53.15}=0.703$$

### 3.2. Raman Analysis

**Figure 3:**
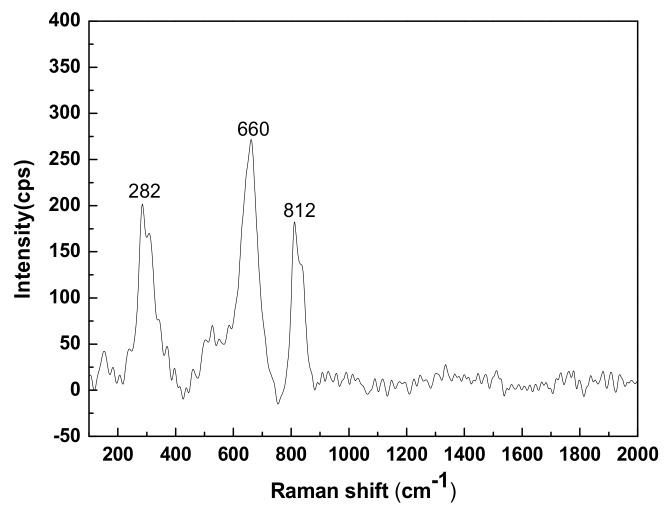
Raman spectra of CNS.

The XRD of CNS revealed that CNS is mainly composed of a fayalite crystalline phase and an amorphous glass phase. The Raman spectra provide a better understanding of the spatial structures of the crystalline and glass phases. According to Figure 3, it is clear that there were three Raman peaks of CNS at 812 cm^−1^ (high wave, 800–1200 cm^−1^), 660 cm^−1^ (middle wave, 600–800 cm^−1^), and 282 cm^−1^ (low wave, <600 cm^−1^).

The Raman band near 850 cm^−1^ is classified as an island-like [SiO_4_]^4−^ tetrahedral structure [26], and it was associated with the Si-O_nonbridging_-Si stretching vibration of the fayalite crystalline phase. The Raman band at 650–750 cm^−1^ was associated with the Si-O_bridging_-Si bending vibration of the glass phase. Its structure consists mainly of [SiO_4_]^4−^ connected to alkaline and alkaline earth cations, but not connected to another Si. The low Raman shift range is the complex mixed stretching and bending vibration of the Si-O_bridging_-Si. This mode is the reticular [SiO_4_]^4−^ tetrahedral structure [27].

### 3.3. Ion Dissolution

**Figure 4:**
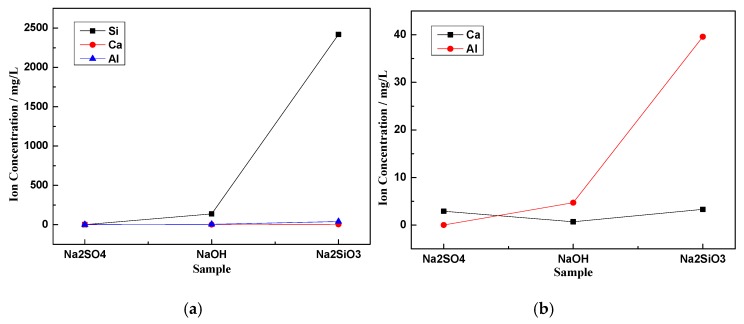
The effects of different activator environments on the dissolution rates of silicon, aluminum, and calcium; (**b**) is a partial enlargement of (**a**).

The activity of CNS is closely related to the characteristics of glass phase dissolution. Figure 4 presents the effects of different activator solutions on the dissolution rates of silicon, aluminum, and calcium in the glass phase of CNS. Figure 4b is a partial enlargement of Figure 4a. According to Figure 4a, the different chemical activators had the greatest influences on Si^4+^ and Al^3+^ dissolution in CNS. When ${\mathrm{Na}}_{2}{\mathrm{SiO}}_{3}$ was used as the activator, the content of Si^4+^ in the leaching solution was the highest at 2419 mg/L due to Si^4+^ in the activator. When NaOH was used as the activator, the content of Si^4+^ in the leaching solution was 137.2 mg/L. However, Si^4+^ underwent the least amount of dissolution in the ${\mathrm{Na}}_{2}{\mathrm{SO}}_{4}$ solution (only 0.36 mg/L). As can be seen from Figure 4b, the dissolution of Al^3+^ in the leaching solutions of ${\mathrm{Na}}_{2}{\mathrm{SO}}_{4}$, NaOH, and ${\mathrm{Na}}_{2}{\mathrm{SiO}}_{3}$ exhibited an increasing trend of 0.01, 4.72, and 39.55 mg/L, respectively. CNS had the least amount of Ca^2+^ in the NaOH leaching solution (only 0.74 mg/L). The ${\mathrm{Na}}_{2}{\mathrm{SiO}}_{3}$ leaching solution had the highest Ca^2+^ content of 3.38 mg/L.

The chemical activators NaOH, ${\mathrm{Na}}_{2}{\mathrm{SO}}_{4}$, and ${\mathrm{Na}}_{2}{\mathrm{SiO}}_{3}$ can break the chemical bonds in the glass phase of CNS. The mechanism is similar to that of GGFBS, which is the depolymerization-condensation reaction of the [SiO_4_]^4−^ tetrahedron and [AlO_4_]^5−^ tetrahedron in the glass phase [28]. In the spatial network structure formed by the [SiO_4_]^4^^−^ tetrahedron and [AlO_4_]^5−^ tetrahedron, Si and Al have high chemical bond energies as network bodies [29]. Given the low bond energy of Ca-O, Ca^2+^ will be dissolved quickly in NaOH, ${\mathrm{Na}}_{2}{\mathrm{SO}}_{4}$, and ${\mathrm{Na}}_{2}{\mathrm{SiO}}_{3}$. Figure 4 reveals that the NaOH and ${\mathrm{Na}}_{2}{\mathrm{SiO}}_{3}$ solutions can promote the breaking of [SiO_4_]^4−^ and [AlO_4_]^5−^ bonds better than the ${\mathrm{Na}}_{2}{\mathrm{SO}}_{4}$ solution, and therefore, can dissolve more Si^4+^ and Al^3+^ than the ${\mathrm{Na}}_{2}{\mathrm{SO}}_{4}$ solution. However, some NaOH will react with Ca^2+^ dissolved in the solution to produce calcium hydroxide, and thus, the NaOH involved in the [SiO_4_]^4−^ and [AlO_4_]^5−^ bond breaking will be reduced greatly. Therefore, ${\mathrm{Na}}_{2}{\mathrm{SiO}}_{3}$ dissolved more Al^3+^ and Ca^2+^ than did NaOH (Table 3).

### 3.4. Effect of Grinding Time on Hydration of ACNCMs

**Figure 5:**
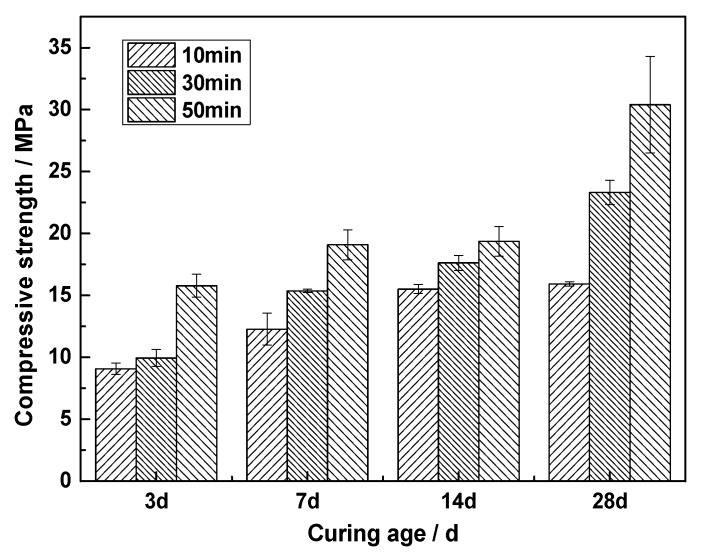
Effects of different grinding times on the hydration of alkali-activated copper and nickel slag cementitious materials (ACNCMs).

The activity index of CNS and the characteristics of glass phase dissolution indicate that CNS has potential cementitious activity. To study the effect of the fineness of CNS on the hydration of ACNCMs, we used a planetary ball mill to grind CNS mechanically for 10, 30, and 50 min, respectively. A laser particle size analyzer characterized the particle size of CNS after grinding. After grinding for 10, 30, and 50 min, the CNS D50 values were 84.58, 55.6, and 8.3 μm, respectively. Figure 5 is a bar chart of the compressive strengths of CNS mixed with 30% slag powder at different grinding times and excited by 7% NaOH solution. With the extension of the grinding time, the compressive strength of ACNCMs at various hydration ages was found to increase. At a curing age of 28 d, the compressive strength of CNS after grinding for 50 min was 30.4 MPa, which was 1.3 times that after grinding for 30 min and 1.9 times that value after grinding for 10 min. Therefore, the extension of the grinding time can promote the hydration of CNS and improve the compressive strength of ACNCMs. This outcome is because mechanical grinding can improve the specific surface area of CNS and increase the heat release of CNS at the beginning of hydration, thereby promoting the participation of CNS in the hydration reaction [30].

### 3.5. Effect of Activators on Hydration of ACNCMs

**Figure 6:**
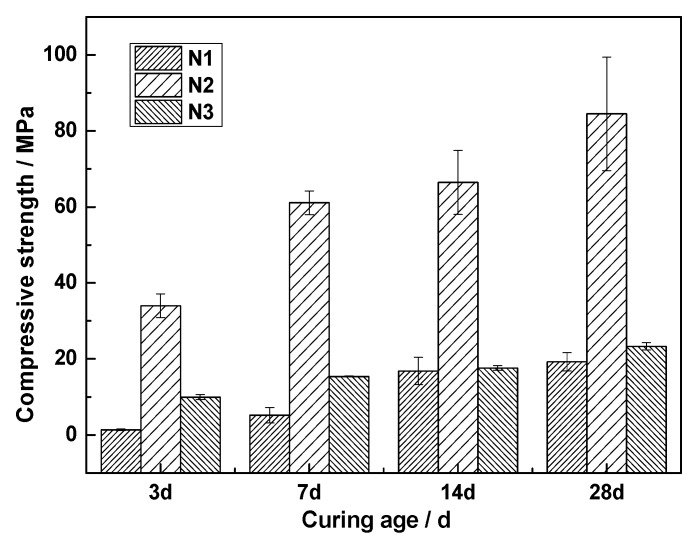
Effects of different activators on the hydration of ACNCMs.

Not only does the mechanical grinding have a great influence on the hydration of slag, but so too does the type of activator. To study the influence of the activator types on the hydration of ACNCMs, we used ${\mathrm{Na}}_{2}{\mathrm{SO}}_{4}$, ${\mathrm{Na}}_{2}{\mathrm{SiO}}_{3}$, and NaOH (respectively corresponding to N1, N2, and N3) in the experiment. It can be determined from Figure 6 that sodium sulfate had the worst excitation effect with a 3-day strength of only 1.4 MPa and a 28-day strength of 19 MPa. When sodium hydroxide was used as the activator, the early strength of ACNCMs developed rapidly, and the strength reached 10 MPa in 3 days. However, the later strength of ACNCMs developed slowly, and the 28-day compressive strength was only 23.3 MPa. The ${\mathrm{Na}}_{2}{\mathrm{SiO}}_{3}$ solution with a modulus of 1 had the best excitation effect with a 3-day compressive strength of 34 MPa and a 28-day compressive strength of up to 84 MPa.

The type of activator has a great influence on the hydration of ACNCMs, which is related to the characteristics of glass phase dissolution in smelting slag. When ${\mathrm{Na}}_{2}{\mathrm{SO}}_{4}$ was used as the activator, CNS had poor glass phase dissolution characteristics and produced fewer hydration products, so the ACNCMs displayed poor macroscopic performance. As an activator, ${\mathrm{Na}}_{2}{\mathrm{SiO}}_{3}$ had the best glass phase dissolution characteristics and produced the most hydration products. Therefore, the ACNCMs displayed good macroscopic performance.

### 3.6. XRD Analyses

**Figure 7:**
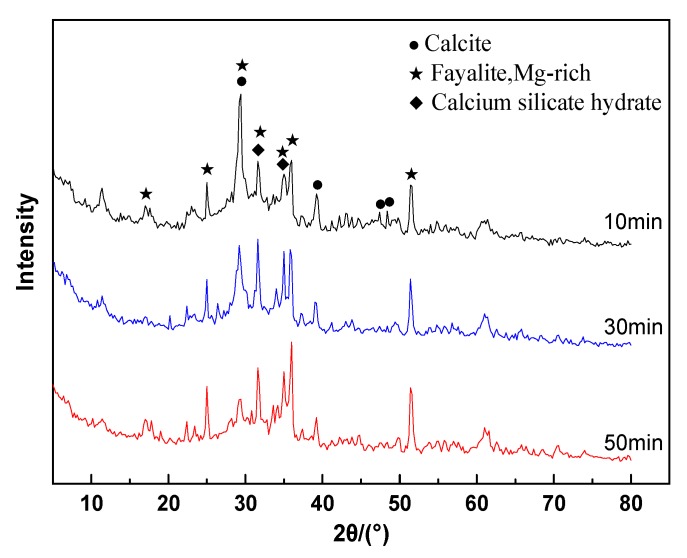
XRD pattern of the hydration products of ACNCMs prepared from CNS with different grinding times.

Figure 7 presents the XRD patterns of the hydration products of ACNCMs prepared by CNS with different grinding times. As compared to Figure 1, it is clear from Figure 7 that ACNCMs not only had the crystalline phase of fayalite (Mg-rich) but also generated new physical-phase calcite and hydrated calcium silicate. The formation of calcite was mainly caused by the chemical reaction of ${\mathrm{CO}}_{2}$ in the air with $H_{2}O$ and Ca^2+^ dissolved from glass in the smelting slag. By comparing the peak strengths of the three groups of samples, with the increase of grinding time, the peak strength of calcite was found to weaken gradually. Meanwhile, the peak strength of hydrated calcium silicate increased gradually. This result indicated that mechanical grinding could promote the hydration reaction of CNS.

**Figure 8:**
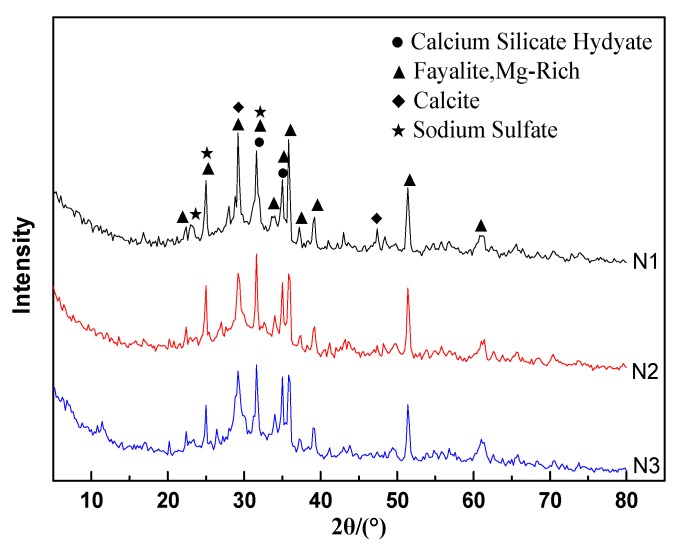
XRD patterns of the hydration products of ACNCMs under different conditions.

Figure 8 presents the XRD patterns of the hydration products of ACNCMs under different activator conditions. Compared to the XRD spectrum presented in Figure 1, the N1, N2, and N3 in Figure 8 presented more new diffraction peaks, except for the peaks containing fayalite. There were diffraction peaks of calcium silicate hydrate and calcite in N1, and there was also a small number of sodium sulfate diffraction peaks. In N2 and N3, there were no sodium sulfate diffraction peaks, and the peak strengths of calcite were significantly less than that in N1, while the peak strengths of hydrated calcium silicate were significantly higher than that in N1. This result indicated that when ${\mathrm{Na}}_{2}{\mathrm{SO}}_{4}$ was used as the activator, a large part of Ca^2+^ in the smelting slag eventually generated calcite, while the amount of generated C-S-H gel was small. Therefore, the excitation effect of ${\mathrm{Na}}_{2}{\mathrm{SO}}_{4}$ was significantly weaker than the effects of ${\mathrm{Na}}_{2}{\mathrm{SiO}}_{3}$ and NaOH. This result was also confirmed by the TG analysis presented in Section 3.8.

### 3.7. FT-IR Analyses

**Figure 9:**
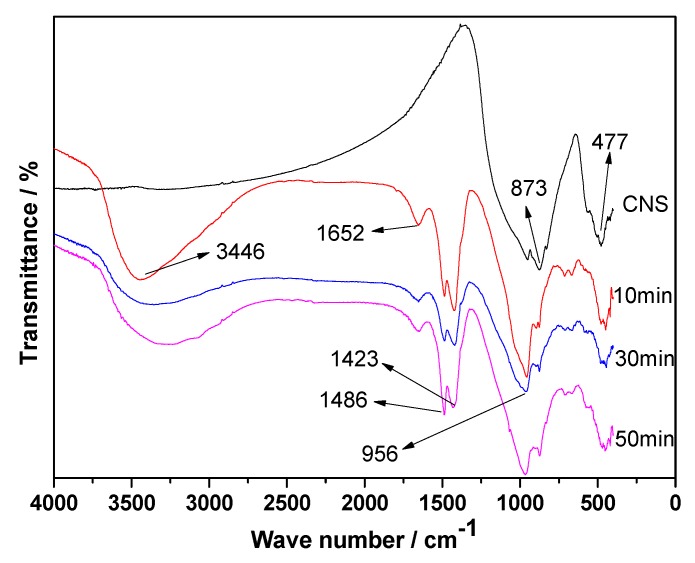
FT-IR spectra of the hydration products of ACNCMs prepared by CNS with different grinding times.

Figure 9 presents the FT-IR (Fourier Transform Infrared Spectrometer) spectra of the hydration products of ACNCMs prepared from CNS with different grinding times. Compared to the CNS, the three groups of samples had more infrared absorption peaks after hydration. The two main absorption peak wavelengths of CNS were 873 and 477 cm^−1^, respectively. The absorption band at 873 cm^−1^ is related to the asymmetric stretching vibration of the ${\mathrm{AlO}}_{4}^{-}$ group [31]. Additionally, 477 cm^−1^ is the bending vibration of Si-O-Si [32]. The absorption peak wavelengths of the ACNCMs hydration products were mainly around 3446, 1652, 1486, 1423, 956, 873, and 477 cm^−1^. Among them, 3446 and 1652 cm^−1^ respectively represented the asymmetric stretching vibration and bending vibration of the combined water −OH group [33]. Additionally, 1486 and 1423 cm^−1^ were classified as ${\mathrm{CO}}_{3}^{2-}$ vibrations [34,35]. Finally, 956 cm^−1^ was attributed to the Si-O stretching vibration in the C-S-H gel hydration product [31,36].

**Figure 10:**
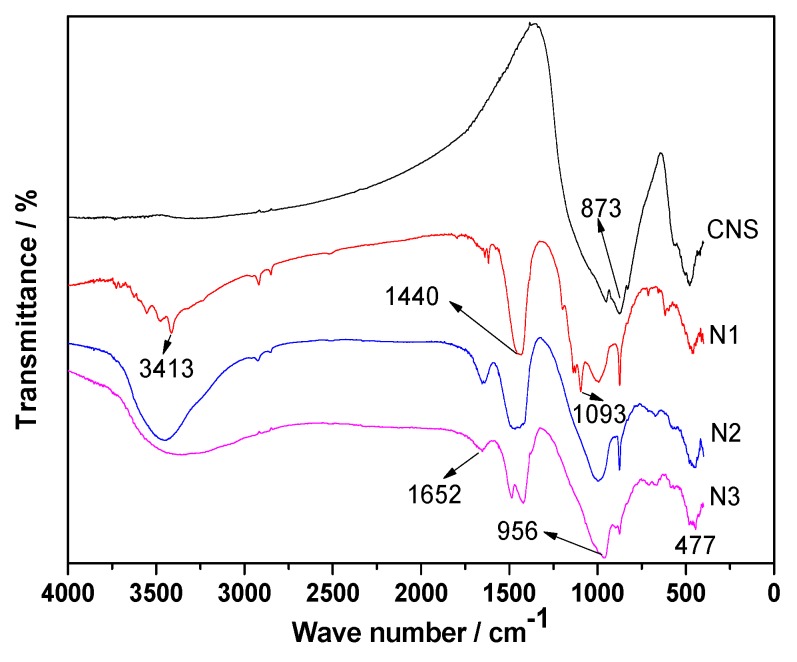
FT-IR analysis of the hydration products of ACNCMs under different excitation conditions.

Figure 10 presents the FT-IR spectra of the hydration products of ACNCMs in different excitation environments. Compared to the original CNS spectra, the infrared absorption peaks of N1, N2, and N3 were significantly different. The two main absorption peak wavelengths of CNS were 873 and 477 cm^−1^, respectively. The absorption band at 873 cm^−1^ was attributed to the asymmetric stretching vibration of the ${\mathrm{AlO}}_{4}^{-}$ group, and that at 477 cm^−1^ it was the bending vibration of Si-O-Si. The wavelengths 3413 and 1652 cm^−1^ respectively represent the asymmetric stretching vibration and bending vibration of the −OH group in water. The absorption peak near 1440 cm^−1^ represents the vibration of ${\mathrm{CO}}_{3}^{2-}$. From the figure, it is clear that the hydration products of N1, N2, and N3 all had more carbonate materials. The absorption peak near the wavelength of 956 cm^−1^ was attributed to the Si−O stretching vibration in the C-S-H gel hydration product, and the peaks of N2 and N3 here were much stronger than that of N1. In addition, compared to N2 and N3, N1 was found to have a small absorption peak at the wavelength of 1093 cm^−1^, which was attributed to the stretching vibration of the S−O bond of the ${\mathrm{SO}}_{4}^{2-}$ group in AFt [32,36].

### 3.8. TG and MIP Analyses

**Figure 11:**
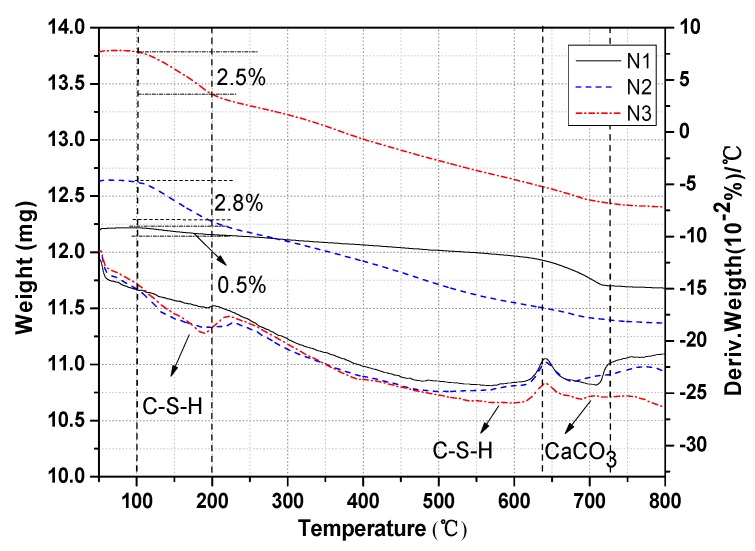
Thermogravimetric analysis of ACNCMs.

Figure 11 presents the TG (Thermogravimetric Analysis) curves of the hydration products of ACNCMs in different activator environments. C-S-H gel dehydration reaction occurs at 100–200 °C [37]. We calculated that the mass loss rate of C-S-H in N1 was 0.5%, that in N2 was 2.8%, and that in N3 was 2.5%. When ${\mathrm{Na}}_{2}{\mathrm{SiO}}_{3}$ was used as the activator, the C-S-H content of the hydration products of ACNCMs was the highest, and it was the lowest when ${\mathrm{Na}}_{2}{\mathrm{SO}}_{4}$ was used as the activator. C-S-H decomposes at 560 °C [38], ${\mathrm{CaCO}}_{3}$ decarburizes at 600 °C [39], and ${\mathrm{CaCO}}_{3}$ decomposition reactions occur at 600–784 °C [40,41]. According to the weight loss of ${\mathrm{CaCO}}_{3}$ revealed in the figure, it was evident that the content of ${\mathrm{CaCO}}_{3}$ in N1 was the highest.

**Figure 12:**
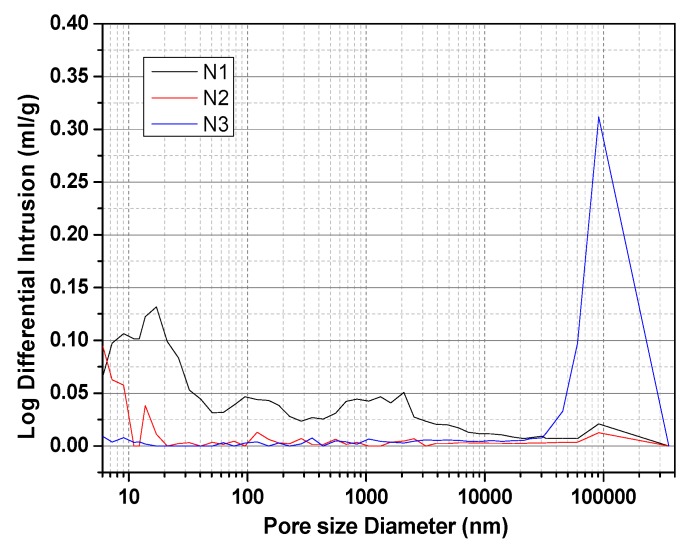
Analysis of the pore structure of ACNCMs.

Figure 12 presents the pore structure diagram of the hydration products of ACNCMs in different activator environments (MIP, Mercury Intrusion porosimetry). As an activator,${\;\mathrm{Na}}_{2}{\mathrm{SO}}_{4}$ has a loose and irregular pore diameter distribution within 10–3000 nm. When NaOH was used as the activator, the pore diameters of the hydration products were larger and distributed at 100 μm. When ${\mathrm{Na}}_{2}{\mathrm{SiO}}_{3}$ was used as the activator, the pore diameters of the hydration products were the smallest and were concentrated within 10 nm. From greatest to least, the porosity values were found to be ${\mathrm{Na}}_{2}{\mathrm{SO}}_{4}$ (37.2%) > NaOH (32.6%) > ${\mathrm{Na}}_{2}{\mathrm{SiO}}_{3}$ (9.7%). Although N1 will generate a small amount of AFt, which reduces the pore size of hydration products, it will increase the porosity of hydration products. It has a lesser effect on strength. When N2 was used as the activator, more of the C-S-H gel hydration products were formed, and they interleaved with each other, thereby reducing the porosity of the structure greatly. This result is beneficial to the compressive strength of ACNCMs.

### 3.9. SEM Analyses

**Figure 13:**
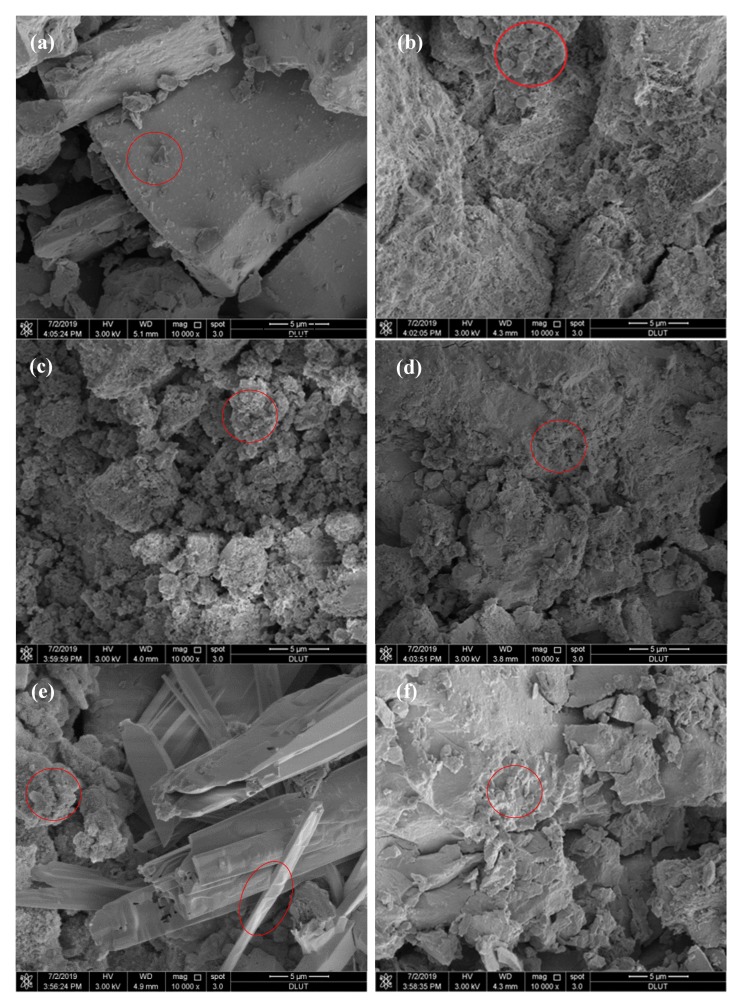
SEM images of different CNS and ACNCMs specimens after hydration for 28 days: (**a**) CNS; (**b**) 10 min grinding; (**c**) 30 min grinding; (**d**) 50 min grinding; (**e**) Na_2_SO_4_ activator; (**f**) Na_2_SiO_3_ activator.

Figure 13 presents the scanning electron microscope (SEM) images of CNS and the ACNCMs composite cementing agent material after hydration for 28 days. It can be seen from Figure 13a that the surface of the CNS was relatively smooth. Compared to Figure 13a, the smooth surface of the CNS was covered with a flocculent substance, as depicted in Figure 13b–f. In addition to the flocculent hydration products, Figure 13f also reveals the existence of some needlelike substances.

When the grinding time was relatively short (such as 10 min), the hydration reaction of CNS was relatively slow, the consumption of the activator NaOH was less, and the excessive NaOH reacted with the calcium ions in the solution to produce $\mathrm{Ca}{\left(\mathrm{OH}\right)}_{2}$, which eventually reacted with the ${\mathrm{CO}}_{2}$ in the air to produce calcite. As revealed by the XRD and FT-IR, the larger the specific surface area of CNS, the more hydrated calcium silicate was generated, and the stronger the absorption peak of the hydrated calcium silicate hydration products of ACNCMs. Additionally, the SEM structure was more compact.

The chemical bonds of the glass-phase [SiO_4_]^4−^ tetrahedron and [AlO_4_]^5^^−^ tetrahedron in CNS and GGFBS were broken when ${\mathrm{Na}}_{2}{\mathrm{SO}}_{4}$, ${\mathrm{Na}}_{2}{\mathrm{SiO}}_{3}$, and NaOH were used as the activators. The three-dimensional network structure with a high degree of polymerization ultimately depolymerized to produce monomers with low degrees of polymerization [20]. The specific depolymerized process is given by Reactions (2) and (3) [26,42]. These monomers with low degrees of polymerization reacted with Ca^2+^ dissolved in the CNS and GGFBS to produce C-S-H with a high degree of polymerization.

(2) $${\left[-\mathrm{Si}-O-\;\mathrm{Si}-\right]}_{n}\;+\;n\left(-\mathrm{OH}\right)\;\to \;\mathrm{nSi}{\left(\mathrm{OH}\right)}_{4}$$

(3) $${\left[-\mathrm{Si}-O-\mathrm{Al}-\right]}_{n}\;+\;n\left(-\mathrm{OH}\right)\;\to \;\mathrm{nAl}{\left(\mathrm{OH}\right)}_{4}^{-}\;+\;\mathrm{nSi}{\left(\mathrm{OH}\right)}_{4}$$

When ${\mathrm{Na}}_{2}{\mathrm{SO}}_{4}$ was used as the activator, the silicon content was very small; thus, the amount of the calcium silicate hydrate hydration product generated by ACNCMs was very small, and most of the Ca reacted with the ${\mathrm{CO}}_{2}$ in the air to produce calcite. Therefore, the macroscopic mechanical performance was poor. In addition, a small amount of Al in the solution reacted with Ca and S to form AFt. The needle-stick AFt filled in the C-S-H gel, and the pore size in the structure was smaller, as verified by FT-IR and MIP. When NaOH was used as the activator, some calcium from the glass phase in the slag reacted with it. Therefore, relatively few C-S-H gels were generated, the pore size in the structure was larger, and the macroscopic mechanical properties were generally good. However, the phase dissolution of slag glass was the best when ${\mathrm{Na}}_{2}{\mathrm{SiO}}_{3}$ was used as the activator. In addition, the activator solution contained a large amount of Si^4+^, so the content of C-S-H in the hydration products of the ACNCMs was the highest. In the pore structure, the pore diameter was small, the SEM structure was more compact, and the macroscopic mechanical properties were the best.

## 4. Conclusions

(1) Mechanical grinding can improve the activity of CNS. The activity indexes of CNS after grinding for 10, 30, and 50 min were found to be 0.662, 0.689, and 0.703, respectively.
(2) Mechanical grinding can promote the hydration reaction of CNS to produce more C-S-H gel, thereby improving the compressive strength of ACNCMs. The 28-day compressive strength of the CNS ground for 50 min was found to be 30.4 MPa, which was 1.3 times that of the CNS ground for 30 min, and 1.9 times that of the CNS ground for 10 min.
(3) CNS is composed of crystalline-phase fayalite and an amorphous glass phase. Fayalite is an island-like [SiO_4_]^4^^−^ tetrahedral structure in which the Si-O chemical bond exists as a non-bridged oxygen bond. The glass phase is a bridged oxygen-connected reticular [SiO_4_]^4^^−^ tetrahedral structure.
(4) ${\mathrm{Na}}_{2}{\mathrm{SO}}_{4}$, NaOH, and ${\mathrm{Na}}_{2}{\mathrm{SiO}}_{3}$ can break the [SiO_4_]^4^^−^ chemical bonds in the glass phase of CNS, but NaOH and ${\mathrm{Na}}_{2}{\mathrm{SiO}}_{3}$ are better at breaking chemical bonds than ${\mathrm{Na}}_{2}{\mathrm{SO}}_{4}$. When ${\mathrm{Na}}_{2}{\mathrm{SO}}_{4}$ was used as the activator, the glass phase dissolution was the worst with less Si^4+^, Al^3+^, and Ca^2+^ in the solution. In contrast, when ${\mathrm{Na}}_{2}{\mathrm{SiO}}_{3}$ was used as the activator, the dissolution characteristics in the glass phase were the best, and the solution contained the most Si^4+^, Al^3+^, and Ca^2+^ with respective concentrations of 2419, 39.55, and 3.38 mg/L.
(5) When ${\mathrm{Na}}_{2}{\mathrm{SO}}_{4}$ was used as the activator, there was very little C-S-H gel generated by the ACNCMs, the macroscopic mechanical performance was the worst, and the 28-day compressive strength was only 19 MPa. When NaOH was used as the activator, there was relatively less C-S-H gel generated by the ACNCMs, and it had a larger pore size and denser structure. The macroscopic mechanical performance was generally good, and the 28-day compressive strength was 23.3 MPa. However, when ${\mathrm{Na}}_{2}{\mathrm{SiO}}_{3}$ was used as the activator, CNS dissolved more Si^4+^ and Ca^2+^ during the hydration reaction, and the ACNCMs generated more C-S-H gel. The pore size was smaller, the structure was more compact, and the macroscopic mechanical performance was better. The 28-day compressive strength was found to be as high as 84 MPa.

## Figures and Tables

**Table 1:** Chemical compositions of copper and nickel slag (CNS) and ground-granulated blast-furnace slag (GGBFS) (%).

Composition	$\;SiO_{2}$	$\;Fe_{2}O_{3}$	$\;Al_{2}O_{3}$	CaO	MgO	$\;SO_{3}$	$Na_{2}O$	NiO	CuO	$Cr_{2}O_{3}$
CNS	32.37	53.87	1.18	1.66	6.53	1.00	0.56	0.54	0.49	0.43
GGFBS	15.43	0.73	19.20	46.27	14.74	---	0.62	---	---	---

**Table 2:** Quantitative analysis of CNS (wt%).

	Rietveld	Spiked	Original
**Amorphous**	0	24.82	27.58
**Fayalite, Mg-rich**	86.70	65.18	72.42
**Zincite**	13.30	10.00	0.000

**Table 3:** Bond energies of elements [29].

Element	Type	Ligancy	M-O Bond Energy (KJ)
Si	Network-forming	4	106
Al	Network-forming	4	101–79
Mg	Intermediate	4	55.5
Ca	Network-modifying	8	32
Na	Network-modifying	6	20

## Data Availability

All data used to support the findings of this study are available from the corresponding authors upon request.
